# Development and Implementation of a Corriedale Ovine Brain Atlas for Use in Atlas-Based Segmentation

**DOI:** 10.1371/journal.pone.0155974

**Published:** 2016-06-10

**Authors:** Kishan Andre Liyanage, Christopher Steward, Bradford Armstrong Moffat, Nicholas Lachlan Opie, Gil Simon Rind, Sam Emmanuel John, Stephen Ronayne, Clive Newton May, Terence John O’Brien, Marjorie Eileen Milne, Thomas James Oxley

**Affiliations:** 1 Vascular Bionics Laboratory, Department of Medicine, Royal Melbourne Hospital, University of Melbourne, Parkville, Victoria, Australia; 2 Department of Radiology, Royal Melbourne Hospital, University of Melbourne, Parkville, Victoria, Australia; 3 Department of Anatomy and Neuroscience, The University of Melbourne, Parkville, Victoria, Australia; 4 The Florey Institute of Neuroscience and Mental Health, University of Melbourne, Parkville, Victoria, Australia; 5 NeuroEngineering Laboratory, Department of Electrical & Electronic Engineering, The University of Melbourne and Centre for Neural Engineering, Parkville, Victoria, Australia; 6 Department of Medicine, Royal Melbourne Hospital, University of Melbourne, Parkville, Victoria, Australia; 7 Faculty of Veterinary and Agricultural Sciences, University of Melbourne, Werribee, Victoria, Australia; College of Mechatronics and Automation, National University of Defense Technology, CHINA

## Abstract

Segmentation is the process of partitioning an image into subdivisions and can be applied to medical images to isolate anatomical or pathological areas for further analysis. This process can be done manually or automated by the use of image processing computer packages. Atlas-based segmentation automates this process by the use of a pre-labelled template and a registration algorithm. We developed an ovine brain atlas that can be used as a model for neurological conditions such as Parkinson’s disease and focal epilepsy. 17 female Corriedale ovine brains were imaged in-vivo in a 1.5T (low-resolution) MRI scanner. 13 of the low-resolution images were combined using a template construction algorithm to form a low-resolution template. The template was labelled to form an atlas and tested by comparing manual with atlas-based segmentations against the remaining four low-resolution images. The comparisons were in the form of similarity metrics used in previous segmentation research. Dice Similarity Coefficients were utilised to determine the degree of overlap between eight independent, manual and atlas-based segmentations, with values ranging from 0 (no overlap) to 1 (complete overlap). For 7 of these 8 segmented areas, we achieved a Dice Similarity Coefficient of 0.5–0.8. The amygdala was difficult to segment due to its variable location and similar intensity to surrounding tissues resulting in Dice Coefficients of 0.0–0.2. We developed a low resolution ovine brain atlas with eight clinically relevant areas labelled. This brain atlas performed comparably to prior human atlases described in the literature and to intra-observer error providing an atlas that can be used to guide further research using ovine brains as a model and is hosted online for public access.

## Introduction

The ovine brain is useful for clinically relevant translational neuroscience research and sheep have been used as a model for neurological diseases because of their relatively large brains (in comparison with rodents), well described somatotopic organization and easily identifiable structures [1]. This allows for easier surgical manipulation, detailed regional analysis and a reduced requirement for higher resolution imaging.

Ovine models have proven to be valuable in the investigation of multiple neurological conditions, allowing for the creation of both functional and neuroanatomical models. A sheep model inducing a persistent Parkinsonian state with the administration of the neurotoxin MPTP has proven to be an efficient translational research model [2]. Furthermore, a Parkinson’s disease treatment study into electrode stimulation of the ovine subthalamic nucleus deemed them as a viable large-animal model for deep brain stimulation side effects [3]. Focal seizure activity visualized in sheep subjects injected with local penicillin in their right prefrontal cortex has provided an effective model for investigating epilepsy with intracranial electroencephalography (EEG) [4]. Subsequent, high-frequency deep brain stimulation of the anterior nucleus of the thalamus resulted in the suppression of the induced spontaneous cortical spike activity in the hippocampus, which was demonstrated to be associated with a therapeutic benefit [5]. Further neurophysiological studies led to the development of an ovine model for Batten disease, supporting long-term longitudinal EEG recordings [6]. Pronuclear injection of human cDNA created a functional model for Huntington’s Disease [7] which is useful for testing of gene therapy based reagents. The similarity in structure and scale between ovine and human brain vasculature (with the exception of the extradural rete mirabilis) allowed for the development of a permanent and transient middle cerebral artery sheep stroke model [8]. Due to its constituent Purkinje Cells, the cerebellum is an area studied for its involvement in coordination of sensory input, motor movement, memory processes and connections with cochlear nuclei [9]. It is of further interest due to its sparing in Batten Disease [10] and its dysplasia in congenital Schmallenberg Virus Infection [11]. We recently reported the use of a cerebral angiography sheep model in the validation of a novel neural interface with the intention of targeting cortical motor areas [12].

Despite the widespread use of the ovine brain in neuroscience research, an MRI atlas of the ovine brain does not exist. We believe that creating an ovine brain atlas would ease further neuroanatomical and functional research using ovine brains as a model. There is growing interest in finding large animal models of human disease [2–6] and identifying the target area on imaging is a process that has been difficult in the past [2–5]. An atlas guides this process to proceed in a much quicker and consistent fashion by the use of a process called segmentation. An MRI atlas would enable researchers to accurately localize different brain areas for better surgical planning and execution and involves a process called segmentation.

Segmentation is the process of partitioning an image where each partition represents a more meaningful subdivision of the whole image. This process of segmentation may be applied to medical imaging in order to select anatomical or pathological areas of interest for further analysis. Although this process is often done manually, it can be automated by the use of image processing computer packages such as ITK-SNAP (Insight Toolkit Snake Automatic Partitioning) [13] and ANTs (Advanced Normalization Tools) [14]. Common methods of achieving automatic segmentation include intensity differentiation [15], and atlas-based segmentation [16]. Atlas-based segmentation is a process that uses a priori anatomical information in the form of a pre-labelled template and a registration algorithm to achieve segmentation [17]. Some of the advantages of automation include the requirement of less anatomical expertise, less time to segment, increased objectivity and possible comparable accuracy to manual segmentation [17].

This research developed and implemented an ovine brain atlas that may be used as a model for neurological conditions such as Parkinson’s disease and focal epilepsy. We demonstrated that atlas based-segmentation is a viable alternative to manual segmentation, with a particular focus on relevant brain structures such as the motor cortices, thalamus, caudate, hippocampus and amygdala.

## Materials and Methods

### Subjects/Apparatus

All experiments were approved by the Howard Florey Institute of Neuroscience and Mental Health Animal Ethics Committee, in accordance with Good Clinical Practices described by the National Health and Medical Research Council.

Adult Corriedale ewes (n = 17) were sedated with intramuscular midazolam (5 mg) followed with induction of anaesthesia using thiopental. Subjects were intubated and anaesthesia was maintained with isoflurane (Mean Alveolar Concentration (MAC) 2.0). All 17 sheep were imaged in a 1.5 Tesla MRI (Signa, GE Medical Systems, Milwaukee, WI, USA) machine using a cardiac coil (FSPGR sequences, Field of View (FOV) 180 mm x 180 mm, slice thickness 1 mm, flip angle 20°, NEX 1). All images were converted to NIFTI (Neuroimaging Informatics Technology Initiative) format for compression and manipulation, including the template and labels.

ANTs [14], was utilised for template construction, non-linear registration and overlap measurements, FSL (FMRIB Software Library) tools were used for linear registration and image manipulation and ITK-SNAP [13] for manual segmentation and creation of mask images. Template construction and registration was performed on a Dell PowerEdge C6145 server with a 64-core AMD Opteron 6282SE 2.6GHz processor and 256GB of RAM.

### Template Creation

**Fig 1:**
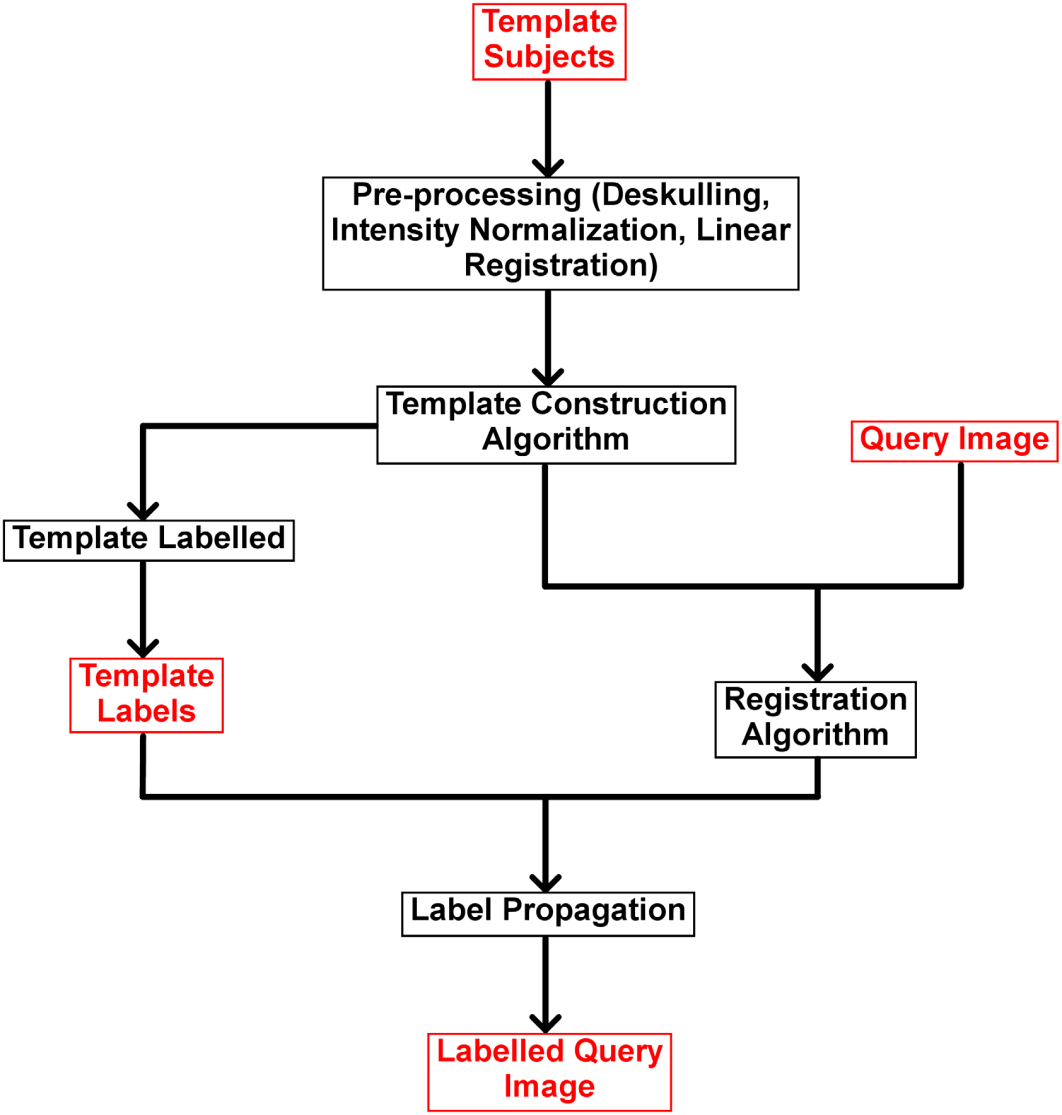
Methods Flowchart. To create a template, an input image (template subject) undergoes a sequence of processing steps (black boxes) such as pre-processing, template construction, labelling and registration to finally output a labelled image. Red boxes represent input and output images.

The process of template creation is shown in Fig 1 and comprises of 5 main parts namely; deskulling, pre-processing, template construction, labelling and registration.

#### Deskulling

Four images were set aside to be used for test purposes while 13 ovine 1.5T MRI images were used for creation of the templates. The 13 1.5T images were run through ANTs bi-directional diffeomorphic template construction algorithm to obtain a skulled template. The similarity metric of cross-correlation (CC) was used with 60 coarse, 90 middle and 30 fine (60x90x30) max-resolution iterations. All other parameters used were kept as default (parallel computation, 0.25 gradient step size, iteration limit of 4, N3BiasFieldCorrection and the Greedy SyN transformation model (GR)). Correspondingly, all 17 1.5T images were also individually deskulled by the combined use of ITK-SNAP and fslmaths. The rough mask created by ITK-SNAP excluded extracortical tissue and was then binarised and expanded by a kernel of 3 mm per pixel. Each individual mask was subtracted from the corresponding image to obtain deskulled images.

#### Pre-processing

Once deskulled, the images were then intensity normalized (fslmaths—inm function; intensity range of 10000) and linearly registered to the largest image with a voxel and image size of 0.5 mm x 0.5 mm x 0.5 mm and 441 mm x 441 mm x 168 mm, respectively. By linearly registering to the largest image dimensions we ensured maximum variation between areas of differing intensities, thereby easing segmentation. The images were manually inspected to ensure identical orientation.

#### Template Construction

The template was formed in accordance with prior research done in atlas-based segmentation [18,19]. Linear registration with FLIRT was chosen due to its previous proven accuracy[20]. The use of ANTS SyN greedy bi-directional diffeomorphic algorithm was used due to it being deemed the most accurate algorithm for registration [20] and template construction [21].

Once the images were linearly registered to each other, the ANTS buildtemplateparallel script was run. Parameters used were the same as those for the skulled template (CC, GR, 60x90x20). No target image was used to ensure that the template was a true population based template. Successively, a low resolution, 1.5T1 weighted population based template was created for use in segmentation.

### Label Creation

Semi-manual segmentation of the low-resolution template was performed by use of the snake tool in ITK-SNAP with each resulting label manually finalized. Isolated areas included both left and right primary motor cortices, ventricles, hippocampus, thalamus, amygdala, hypothalamus and cerebellum (Table 1). These areas were chosen to represent relevant targets in neuroscience research that uses ovine brains as a model. The ventricles were chosen for segmentation as a reference due to their consistency across subjects. Labelling was preferentially done on a low resolution template because most published ovine brain MRIs are obtained at 1.5 Tesla [4,5,8]. The test subject population was also manually segmented in the same process as the template.

**Table 1:** Target Areas.

Areas	Potential Model	References
Motor Cortex	Electrocorticography, Motor Mechanism, Bionic Limbs	Oxley et al. [12], Simpson and King [22], Bagley [23], Grovum and Gonzalez [24]
Somatosensory Cortex	Electrocorticography, Mechanosensory Feedback	Johnson et al. [1], Gierthmuehlen et al. [25]
Caudate, Substantia Nigra	Parkinson’s Disease	Baskin et al. [2]
Subthalamic Nucleus, Hippocampus, Thalamus	Deep Brain Stimulation	Lentz et al. [3], Stypulkowski et al. [5]
Amygdala	Epilepsy	Opdam et al. [4]
Cerebellum	Coordination, Batten Disease, Schmallenberg Viral Infection	Salouci et al. [9], Sawiak et al. [10], Peperkamp et al. [11]
Olfactory Lobes	Neurogenesis	Brus et al. [26]

Previous studies using ovine brains as models have focussed on particular regions for neurological conditions. The use of labelling as a part of an atlas may facilitate improvements in the use of such models.

### Registration

Registration was performed with the use of ANTs rigid linear and non-linear Greedy SyN transformation model, a bi-directional diffeomorphic deformation. Warp files generated from the registration of the skulled template to the skulled test subject MRIs were applied to the deskulling label creating a transformed label specific to the target image vector space. Similarly, warp files of the transformation from the deskulled template to the manually deskulled test subjects were applied to the template labels to obtain the segmented labels.

Similarity metrics were calculated using ANTs LabelOverlapMeasures with results reported as Dice Similarity Coefficients (DSC), Jaccard Coefficients, False Positive and False Negative values (Table 2).

**Table 2:** Segmentation Metrics.

Segmentation Metric	Formula
Mean/Dice Similarity Coefficient (MO)	$\text{MO }=\;2\frac{{\sum^{\text{​}}}_{\text{r}}\left|{\text{S}}_{\text{r}}{\cap^{\text{​}}}^{\text{​}}{\text{T}}_{\text{r}}\right|}{{\sum^{\text{​}}}_{\text{r}}\left(\left|{\text{S}}_{\text{r}}\right|+\left|{\text{T}}_{\text{r}}\right|\right)}$
Union Overlap/Jaccard Coefficient (UO)	$\text{UO }=\;\frac{{\sum^{\text{​}}}_{\text{r}}\left|{\text{S}}_{\text{r}}{\cap^{\text{​}}}^{\text{​}}{\text{T}}_{\text{r}}\right|}{{\sum^{\text{​}}}_{\text{r}}\left|{\text{S}}_{\text{r}}{\cup^{\text{​}}}^{\text{​}}{\text{T}}_{\text{r}}\right|}$
False Negative (FN)	$\text{FN }=\;\frac{{\sum^{\text{​}}}_{\text{r}}\left|{\text{T}}_{\text{r}}\backslash {\text{S}}_{\text{r}}\right|}{{\sum^{\text{​}}}_{\text{r}}\left|{\text{T}}_{\text{r}}\right|}$
False Positive (FP)	$\text{FP }=\;\frac{{\sum^{\text{​}}}_{\text{r}}\left|{\text{S}}_{\text{r}}\backslash {\text{T}}_{\text{r}}\right|}{{\sum^{\text{​}}}_{\text{r}}\left|{\text{S}}_{\text{r}}\right|}$
Volume Similarity/Relative Volume Difference (VS)	$\text{VS }=\;2\frac{{\sum^{\text{​}}}_{\text{r}}\left(\left|{\text{S}}_{\text{r}}\right|-\;\left|{\text{T}}_{\text{r}}\right|\right)}{{\sum^{\text{​}}}_{\text{r}}\left(\left|{\text{S}}_{\text{r}}\right|+\left|{\text{T}}_{\text{r}}\right|\right)}$
Distance Error (DEr)	${\text{DE}}_{\text{r}}\;=\;\frac{1}{\text{P}}\sum_{\text{p}=1}^{\text{p}}\text{mindist }\left({\text{S}}_{\text{r}}{\text{B}}_{\text{p}},{\text{T}}_{\text{r}}\text{B}\right)$ where mindist = minimum distance from source region boundary point SrBp to entire target region boundary TrB.
Spatial Extent (SE)	SE = vol(P>t) 0≤SE≤V where $\text{P}\left(\text{x},\text{y},\text{z}\right)\;=\;\frac{1}{\text{N}}\sum_{\text{i}=1}^{\text{N}}{\text{L}}_{\text{i}}\left(\text{x},\text{y},\text{z}\right)$

Sr = Source Region (Image 1), Tr = Target Region (Image 2). There are several metrics for measuring overlap of two 3-dimensional images. Most studies focus on the Dice Similarity Coefficient or Mean Overlap. Due to its ubiquitous use this study will also use it as the main metric of comparison.

The whole process including atlas construction and manual labelling was performed twice to create labels that would be the most accurate (as determined by the segmentation metrics). Subsequently, segmentations were performed twice, with each manual label compared with each segmented label respectively with the results averaged and reported. The template labels which obtained the most accurate segmentation were then selected to be part of the final atlas. Intra-observer variability was calculated by comparing both sets of manual segmentation to each other.

## Results

### Ovine Brain Atlas

**Fig 2:**
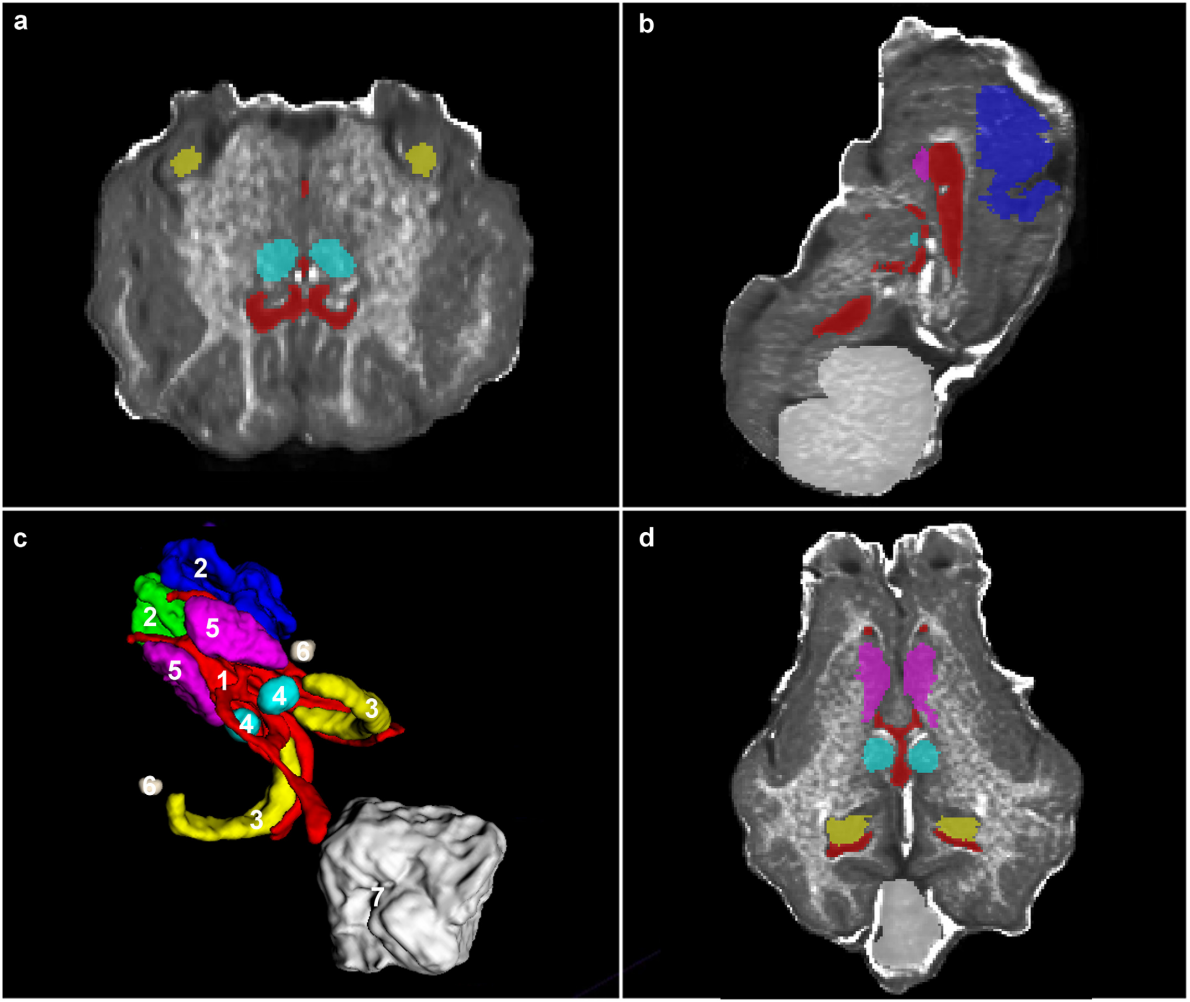
Atlas—Template and Associated Labels. Segmented Atlas showing (1) Ventricles, (2) Motor Cortices, (3) Hippocampi, (4) Thalami, (5) Caudate (6) Amygdala and (7) Cerebellum illustrating the (a) transverse, (b) parasagittal, (d) dorsal planes and (c) 3d rendering of labels on a ventrolateral view with rostral to the left of the image.

The ovine atlas set is composed of the low resolution template (Fig 2a, 2b and 2d) and its associated labels (Fig 2c).

### Deskulling Algorithm

**Fig 3:**
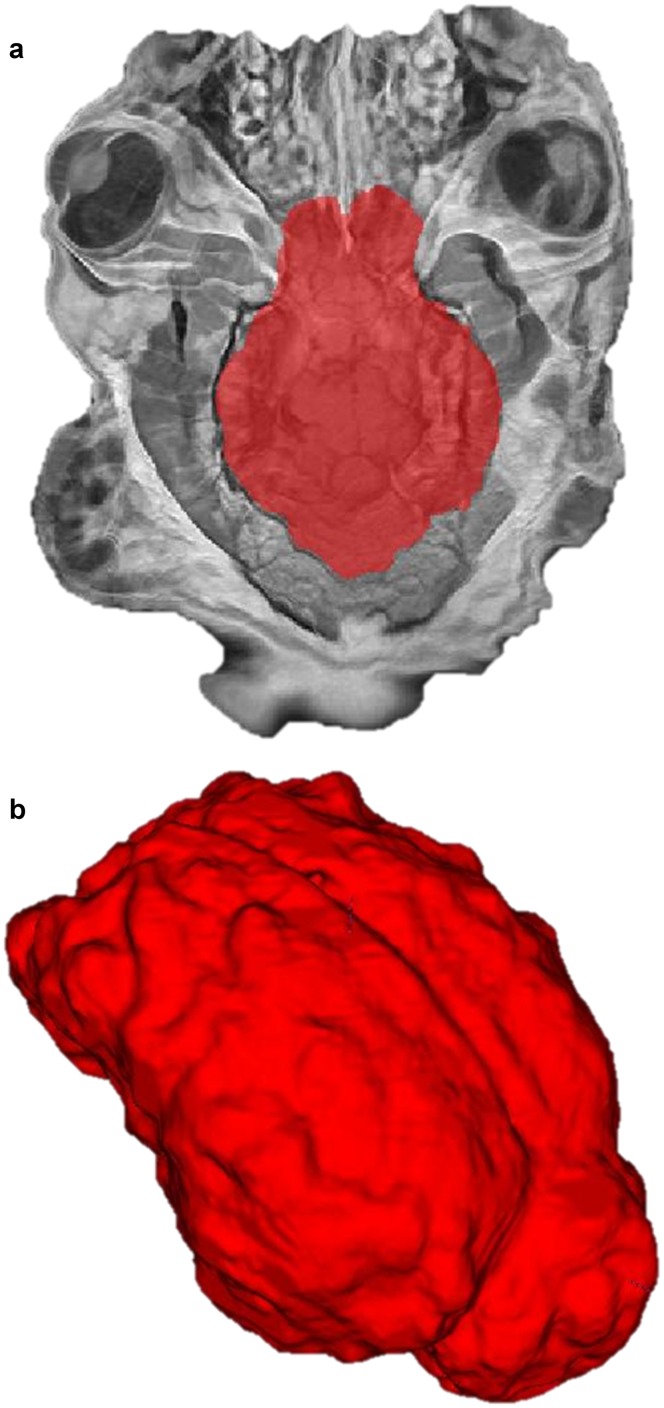
The (a) skulled template and (b) deskulling mask. Variability displayed in extracortical areas reinforces the reason why deskulling is an important step in pre-processing.

The deskulling algorithm makes use of the skulled ovine low resolution template (Fig 3a), the deskulling mask (Fig 3b) and ANTS non-linear registration. Automatic deskulling was accurate with an average dice similarity coefficient of 0.87 when compared with manual deskulling (Table 3).

**Table 3:** Deskulling Metrics.

Subject	Jaccard Coefficient	DICE Coefficient	False Negative	False Positive
**1**	0.804552	0.891692	0.109895	0.106716
**2**	0.746937	0.855139	0.0610956	0.214904
**3**	0.764086	0.866269	0.0954717	0.168886
**4**	0.7673	0.86833	0.141702	0.121401

The table shows the high accuracy of the skulled template and deskulling mask. Ideal values of Jaccard and Dice Similarity Coefficients are 1 (complete overlap) and ideal values of False Negative and False Positive Values are 0 (no voxels outside of the overlap area).

### Atlas-Based Segmentation

**Fig 4:**
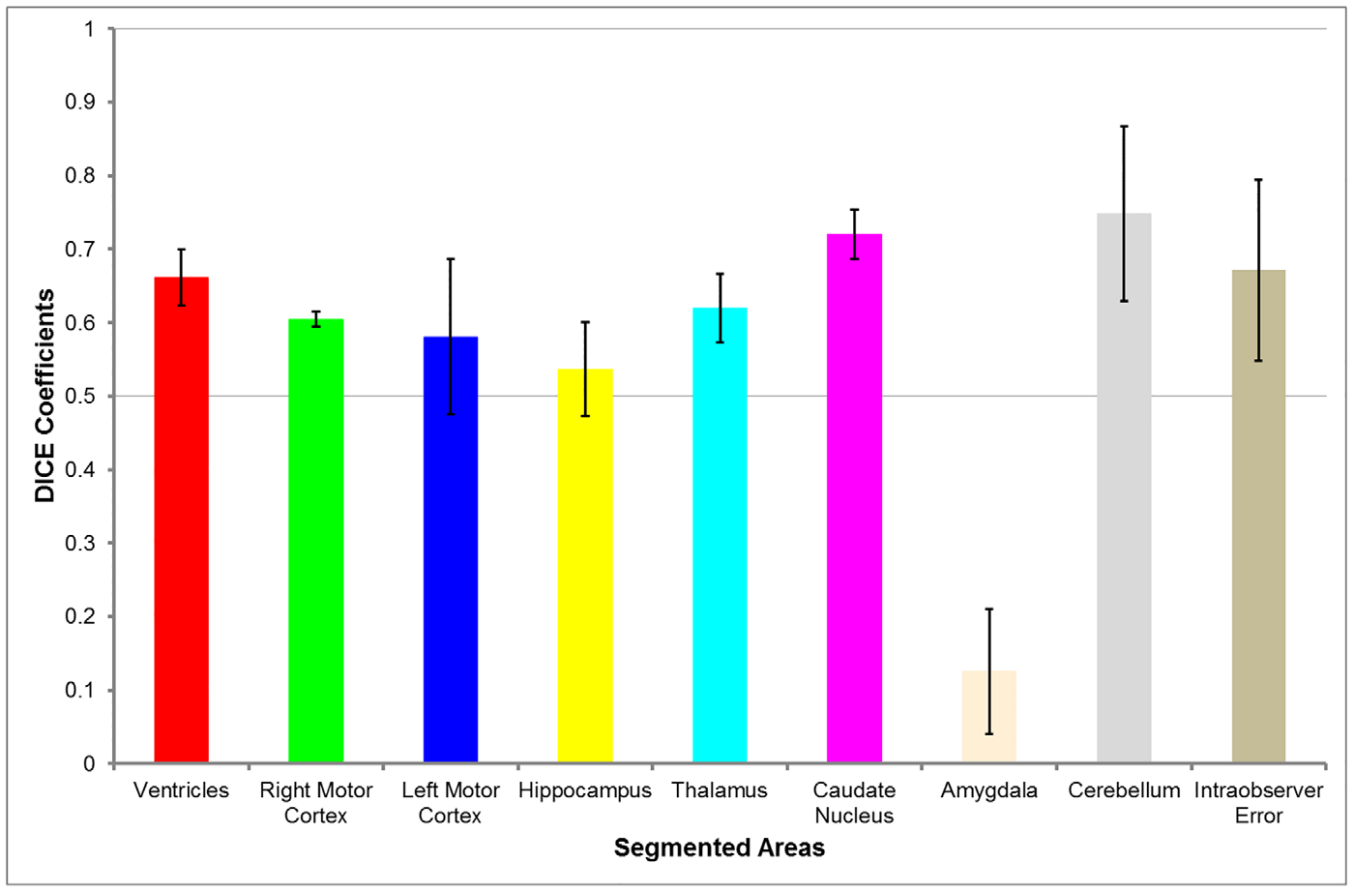
Dice Similarity Coefficients of all structures in the low resolution template. Values range from 0.12 for the amygdala to 0.75 for the cerebellum. Red = Ventricles, Green = Right Motor Cortex, Dark Blue = Left Motor Cortex, Yellow = Hippocampus, Light Blue = Thalamus, Purple = Caudate Nucleus, Peach = Amygdala, Grey = Cerebellum, Brown = Intraobserver Error. Error bars show the standard deviation of the mean for all compared structures. Intraobserver Error was calculated by comparing manual segmentations of all seven labelled structures in the four test subjects. Number of test subjects (n = 4).

Results of the atlas-based segmentation differ for each structure with our model demonstrating a high degree of similarity for the caudate nuclei (0.72), thalami (0.62) and cerebellum (0.75) while considering intra-observer error (Fig 4). The right motor cortices (0.60), left motor cortices (0.58) and hippocampus (0.54) had lower dice similarity coefficients than the caudate, thalami and cerebellum whereas the amygdala was found to have the lowest Dice Similarity Coefficient (0.13).

**Fig 5:**
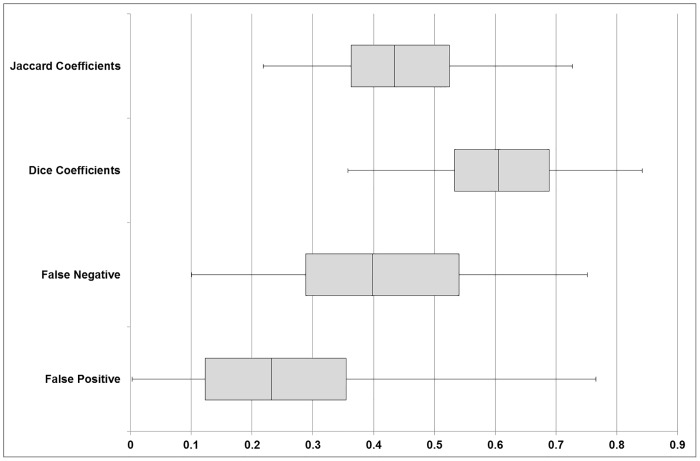
Box plot displaying the average of all comparisons performed across all segmented structures. Shows the median Dice Similarity Coefficient to be 0.61 with the False Positive and False Negative values having a very large variation. This indicates a wide spread in the accuracy of segmentation with regards to various structures. The Jaccard Coefficient is as expected lower than the Dice proportionally due to single use of the intersect in its calculation leading to a more precise comparison.

Overlap measures summarising the comparison of all segmented structures between atlas-based and manual segmentation labels (Fig 5) identify the median of Dice Similarity Coefficients to be 0.61 (range 0.53 to 0.69). The Jaccard Coefficients were less accurate with a median of 0.43 (range 0.36 to 0.53). There was a wide range in both false negative (0.29 to 0.54) and false positive (0.12–0.36) values.

## Discussion

Here we present the development of a MRI-Ovine Brain Atlas that can assist in easy isolation of ovine brain structures and will be a valuable tool in continuing neuroscience research. The low resolution ovine brain atlas performed accurately and consistently in comparison to previous studies and to measured intra-observer error.

### Atlas-Based Segmentation

As shown by the high dice coefficients (DSC 0.58–0.75) (Fig 4), template based segmentation is comparable to manual segmentation in isolating certain brain structures. Structures such as the ventricles, thalamus caudate and cerebellum were the most accurate in the segmentation (DSC 0.62–0.75) likely due to their uniform location, consistent volume and large intensity differentiation with regards to surrounding structures. These coefficients are comparable to intra-observer error (DSC 0.67) and previous segmentation described in the literature (DSC 0.56–0.91) [17,20,21]. This not only supports the use of the template and its associated labels but also concurrently the ANTS registration algorithm. Though no other registration algorithms were tested in the study, it is possible that the overlap results could be improved with the use of other volume registration methods such as ART [27] or surface registration methods such as FreeSurfer [28]. However, a study [29] comparing the two has recommended that the algorithm used to perform the template construction should be used for registration as it maximises accuracy, further supporting the use of ANTS with this ovine brain atlas.

Surprisingly, the motor cortices were segmented more accurately than expected (DSC 0.58–0.60), comparable to intra-observer error (DSC 0.67). The ovine motor cortex is defined as the superior frontal convolution bordered by the medial longitudinal fissure in the midline, the ansate sulci posteriorly and lateral sulci laterally [22]. However, since its inferior border hasn’t been concisely defined, we extended it to the most inferior aspect of grey matter close to the superior border of the lateral ventricles to encapsulate all areas that it could possibly be situated in. The relative accuracy in segmentation suggests that Corriedale sheep cortical sulci and gyri are consistent enough for atlas-based segmentation, allowing for research in electrocorticography. Furthermore, this consistency could be tested with regards to the ovine somatosensory cortices [1], another subject of electrocorticography research [25], identifying another useful cortical target for segmentation.

The amygdala was a difficult structure to segment (DSC 0.13) due to its poor intensity differentiation from the surrounding structures and lack of known surrounding anatomical landmarks. The use of the most anterior border of the hippocampus was as a reference point resulted in variability in the rostrocaudal and mediolateral directions. However, if the amygdala location could be localized accurately in this template, the transformation would ensure that the amygdala location would be more accurate than that currently obtained in this study.

The variability of our dice coefficients, ranging from 0.13 for the amygdala to 0.75 for the cerebellum, elucidates factors which lead to low and high similarity coefficients respectively. Low similarity coefficients are obtained when the structure to be segmented has poor voxel intensity differentiation from surrounding structures [13,17], poorly demarcated anatomical landmarks and high anatomical variability from subject to subject [17]. Though the lack of clearly defined edges due to low voxel intensity differences cannot be addressed by manual or semi-automatic segmentation, if the area is labelled accurately on an atlas the accuracy will be reflected in the segmentation. Anatomical landmarks surrounding the structure can be rigorously studied leading to better definition of the area to be segmented, further improving similarity coefficients. To improve the inaccuracy due to the high anatomical variability there are a few methods one can pursue, such as the inclusion of more subjects in the atlas, or the creation of multiple atlases with each different anatomical variant labelled [17].

### Deskulling Algorithm

Atlas-based segmentation of brain structures is best performed on deskulled images since it aids volume registration [29]. This is particularly relevant in ovine MRIs since a large proportion of the scan includes extracortical brain matter which has to be removed before processing can be done on the images. Deskulling can be done manually, but this is time-consuming and variable especially if there are many subjects for testing. The skulled atlas and deskulling mask performed extremely well (DSC 0.85–0.89) (Table 3) in comparison to intra-observer error and selected brain areas. Though some degree of post-segmentation processing was required, likely due to the variability of extraneous brain matter such as the nasal cavity and facial muscles, this was minimally time-consuming in comparison to deskulling manually. Further improvement in the deskulling template could be achieved by focussing the registration on the rigid ovine skull, a more consistent surface, rather than on the entire brain, thus negating the variability from the extracortical brain matter.

### Ovine Brain Atlas

Multiple steps were taken to ensure that the template created was an average of all subjects and not dependant on one single subject. Firstly, since the population of sheep are all of identical species and of similar age, there is a high structural correspondence between the images. By not setting a main subject in ANTS template building algorithm, bias dependent on topological idiosyncrasy of any individual brain was avoided ensuring that the template was a true population average. Finally, by avoiding the use of manual landmarking and normalizing the origin point to zero (0, 0, 0) we prevented the image from being driven by a specific anatomical structure and ensured that the template shape was independent of any individuals’ anatomical coordinate system.

### Comparison of Overlap Measures

Similarity metrics compare the degree of overlap of two binary labels. DSC is the most popular [20] and measures the amount of overlap in the intersection of the atlas label and query label divided by the sum of the individual areas. This measurement includes the overlap area twice as part of the source region and target region and therefore overestimates the amount of overlap. In comparison, the Jaccard Coefficient is similar in that it compares the overlap of the intersection of two labels divided by the total area, not counting the intersect twice. Both the DSC and Jaccard will have values ranging from 0 to 1, with no overlap represented by 0 and complete overlap represented by 1.

False negative overlap refers to the amount of voxels in a volume that are incorrectly labelled compared with false positive overlap which measures how many voxels outside a volume are incorrectly labelled. Both false positive and false negative metrics have values ranging from 0 to 1. In a perfect situation both false positive and false negative values will be 0.

Based on their methods of comparison, the Dice similarity metric is comparable to the Jaccard coefficient and the false negative metric is comparable to the false positive metric. However, all metrics measure overlap as a product of the total area and therefore are comparable to each other. A Tukey statistical significance test was performed and supported the similarities between the similarity metrics when compared with each other (S1 Text). The exception to this was the comparison of the second atlas to the first set of manual segmentations which found that false negative was significantly different to the other similarity metrics.

### Limitations

A limitation of the study was the inclusion of only one ovine breed rather than a variety, especially those used in other studies such as Polypay mixed breeds [5], Merino [4] and Rambuillett [2] types. By excluding other ovine breeds, the generalisability of the atlas’s accuracy was limited to the Corriedale breed. Though the atlas is still applicable, the results of the segmentation are dependent on structural differences between ovine breeds. There has been no published research done into the anatomical variation between different ovine breed brains and therefore an atlas cannot be conclusively defined as all-encompassing until segmentation studies are done across separate sheep breeds. The degree of heterogeneity in brain anatomy between different ovine breeds represents a potential avenue for future investigation.

Previous human atlases were created with a standard coordinate system such as Talaraich [30] or MNI [31] to create a spatial reference system for image analysis. We avoided landmarking and normalizing our ovine brain atlas to a specific reference point to obtain a more accurate registration. However as a result the atlas created was not standardized in accordance with previous reference systems. Though this has no negative effects on the segmentation, future studies might find it useful to create a standard space to help with referencing.

In the future, the ovine brain atlas may be expanded by labelling more structures, and the existing structures could be more accurately defined based on ovine neuroanatomical research. The inclusion of other ovine breeds would also contribute to the comprehensiveness of the atlas and would be another aspect to explore. The standardization of the atlas by its transposition to a standardised spatial reference system could assist with future referencing of ovine brain structures. The addition of 7T scans would enable the creation of a high-resolution template which could be useful for cortical segmentation. Finally, testing the atlas on different segmentation methods to isolate the most accurate registration algorithm could be another endeavor to undertake.

## Conclusion

A low resolution ovine brain atlas was developed with several clinically relevant areas labelled. On testing, the low resolution atlas performed comparably to prior atlases described in the literature and to intra-observer error, suggestive of its utility as an atlas that can be used to guide further research using an ovine brain model. Atlas-based segmentation by the use of this atlas is a viable alternative to manual segmentation especially with regards to the motor cortices, thalamus, caudate and cerebellum. In the process of creating an ovine atlas, a deskulling mask and template was developed which on implementation performed remarkably compared with the ovine brain atlas, providing a more time efficient and consistent method of deskulling ovine brain MRIs. Our atlas is hosted on GitHub [32], alongside scripts used for automatic segmentation and deskulling, and is free to use for academic or commercial purposes (with appropriate attribution).

## Supporting Information

S1 Data Overlap Measures. (XLSX)

S1 Text Statistic Comparison. (DOCX)
