# Supplementary material for: Development and Implementation of a Corriedale Ovine Brain Atlas for Use in Atlas-Based Segmentation
Source: PLoS One. 2016 Jun 10;11(6):e0155974. doi: 10.1371/journal.pone.0155974 (PMC4902240; doi:10.1371/journal.pone.0155974)
Supplement: S1 Text — (DOCX) [file pone.0155974.s002.docx]

**One-way ANOVA: T2M2Value versus Template2 Manual 2**

Method

Null hypothesis All means are equal

Alternative hypothesis At least one mean is different

Significance level α = 0.05

Equal variances were assumed for the analysis.

Factor Information

Factor Levels Values

Template2 Manual 2 4 DICE Coefficient, False Negative, False Positive, Jaccard Coefficient

Analysis of Variance

Source DF Adj SS Adj MS F-Value P-Value

Template2 Manual 2 3 0.4792 0.15974 3.57 0.017

Error 108 4.8354 0.04477

Total 111 5.3146

Model Summary

S R-sq R-sq(adj) R-sq(pred)

0.211594 9.02% 6.49% 2.15%

Means

Template2 Manual 2 N Mean StDev 95% CI

DICE Coefficient 28 0.5191 0.1962 (0.4398, 0.5983)

False Negative 28 0.4826 0.2200 (0.4034, 0.5619)

False Positive 28 0.3741 0.2562 (0.2948, 0.4533)

Jaccard Coefficient 28 0.3712 0.1630 (0.2919, 0.4504)

Pooled StDev = 0.211594

**Tukey Pairwise Comparisons**

Grouping Information Using the Tukey Method and 95% Confidence

Template2 Manual 2 N Mean Grouping

DICE Coefficient 28 0.5191 A

False Negative 28 0.4826 A B

False Positive 28 0.3741 A B

Jaccard Coefficient 28 0.3712 B

Means that do not share a letter are significantly different.

**Tukey Simultaneous 95% CIs**

**Interval Plot of T2M2Value vs Template2 Manual 2**

**One-way ANOVA: T2M1Value versus Template2 Manual 1**

Method

Null hypothesis All means are equal

Alternative hypothesis At least one mean is different

Significance level α = 0.05

Equal variances were assumed for the analysis.

Factor Information

Factor Levels Values

Template2 Manual 2 4 DICE Coefficient, False Negative, False Positive, Jaccard Coefficient

Analysis of Variance

Source DF Adj SS Adj MS F-Value P-Value

Template2 Manual 2 3 2.066 0.68876 14.24 0.000

Error 108 5.223 0.04836

Total 111 7.290

Model Summary

S R-sq R-sq(adj) R-sq(pred)

0.219917 28.35% 26.36% 22.94%

Means

Template2 Manual 2 N Mean StDev 95% CI

DICE Coefficient 28 0.4393 0.2129 (0.3569, 0.5217)

False Negative 28 0.6444 0.2095 (0.5620, 0.7267)

False Positive 28 0.3215 0.2750 (0.2391, 0.4039)

Jaccard Coefficient 28 0.3034 0.1691 (0.2210, 0.3858)

Pooled StDev = 0.219917

**Tukey Pairwise Comparisons**

Grouping Information Using the Tukey Method and 95% Confidence

Template2 Manual 2 N Mean Grouping

False Negative 28 0.6444 A

DICE Coefficient 28 0.4393 B

False Positive 28 0.3215 B

Jaccard Coefficient 28 0.3034 B

Means that do not share a letter are significantly different.

**Tukey Simultaneous 95% CIs**

**Interval Plot of T2M1Value vs Template2 Manual 1**

**One-way ANOVA: T1M2Value versus Template1 Manual 2**

Method

Null hypothesis All means are equal

Alternative hypothesis At least one mean is different

Significance level α = 0.05

Equal variances were assumed for the analysis.

Factor Information

Factor Levels Values

Template1 Manual 2 4 DICE Coefficient, False Negative, False Positive, Jaccard Coefficient

Analysis of Variance

Source DF Adj SS Adj MS F-Value P-Value

Template1 Manual 2 3 0.3312 0.11041 1.89 0.136

Error 108 6.3191 0.05851

Total 111 6.6503

Model Summary

S R-sq R-sq(adj) R-sq(pred)

0.241888 4.98% 2.34% 0.00%

Means

Template1 Manual 2 N Mean StDev 95% CI

DICE Coefficient 28 0.5049 0.2229 (0.4143, 0.5955)

False Negative 28 0.4797 0.2884 (0.3891, 0.5703)

False Positive 28 0.4358 0.2706 (0.3452, 0.5264)

Jaccard Coefficient 28 0.3616 0.1673 (0.2710, 0.4522)

Pooled StDev = 0.241888

**Tukey Pairwise Comparisons**

Grouping Information Using the Tukey Method and 95% Confidence

Template1 Manual 2 N Mean Grouping

DICE Coefficient 28 0.5049 A

False Negative 28 0.4797 A

False Positive 28 0.4358 A

Jaccard Coefficient 28 0.3616 A

Means that do not share a letter are significantly different.

**Tukey Simultaneous 95% CIs**

**Interval Plot of T1M2Value vs Template1 Manual 2**

**One-way ANOVA: T1M2Value versus Template1 Manual1**

Method

Null hypothesis All means are equal

Alternative hypothesis At least one mean is different

Significance level α = 0.05

Equal variances were assumed for the analysis.

Factor Information

Factor Levels Values

Template1 Manual1 4 DICE Coefficient, False Negative, False Positive, Jaccard Coefficient

Analysis of Variance

Source DF Adj SS Adj MS F-Value P-Value

Template1 Manual1 3 0.3312 0.11041 1.89 0.136

Error 108 6.3191 0.05851

Total 111 6.6503

Model Summary

S R-sq R-sq(adj) R-sq(pred)

0.241888 4.98% 2.34% 0.00%

Means

Template1 Manual1 N Mean StDev 95% CI

DICE Coefficient 28 0.5049 0.2229 (0.4143, 0.5955)

False Negative 28 0.4797 0.2884 (0.3891, 0.5703)

False Positive 28 0.4358 0.2706 (0.3452, 0.5264)

Jaccard Coefficient 28 0.3616 0.1673 (0.2710, 0.4522)

Pooled StDev = 0.241888

**Tukey Pairwise Comparisons**

Grouping Information Using the Tukey Method and 95% Confidence

Template1 Manual1 N Mean Grouping

DICE Coefficient 28 0.5049 A

False Negative 28 0.4797 A

False Positive 28 0.4358 A

Jaccard Coefficient 28 0.3616 A

Means that do not share a letter are significantly different.

**Tukey Simultaneous 95% CIs**

**Interval Plot of T1M2Value vs Template1 Manual1**

**One-way ANOVA: T2M2Value versus Template2 Manual 2**

Method

Null hypothesis All means are equal

Alternative hypothesis At least one mean is different

Significance level α = 0.05

Equal variances were assumed for the analysis.

Factor Information

Factor Levels Values

Template2 Manual 2 4 DICE Coefficient, False Negative, False Positive, Jaccard Coefficient

Analysis of Variance

Source DF Adj SS Adj MS F-Value P-Value

Template2 Manual 2 3 0.4792 0.15974 3.57 0.017

Error 108 4.8354 0.04477

Total 111 5.3146

Model Summary

S R-sq R-sq(adj) R-sq(pred)

0.211594 9.02% 6.49% 2.15%

Means

Template2 Manual 2 N Mean StDev 95% CI

DICE Coefficient 28 0.5191 0.1962 (0.4398, 0.5983)

False Negative 28 0.4826 0.2200 (0.4034, 0.5619)

False Positive 28 0.3741 0.2562 (0.2948, 0.4533)

Jaccard Coefficient 28 0.3712 0.1630 (0.2919, 0.4504)

Pooled StDev = 0.211594

**Tukey Pairwise Comparisons**

Grouping Information Using the Tukey Method and 95% Confidence

Template2 Manual 2 N Mean Grouping

DICE Coefficient 28 0.5191 A

False Negative 28 0.4826 A B

False Positive 28 0.3741 A B

Jaccard Coefficient 28 0.3712 B

Means that do not share a letter are significantly different.

**Tukey Simultaneous 95% CIs**

**Interval Plot of T2M2Value vs Template2 Manual 2**

**One-way ANOVA: T2M1Value versus Template2 Manual 1**

Method

Null hypothesis All means are equal

Alternative hypothesis At least one mean is different

Significance level α = 0.05

Equal variances were assumed for the analysis.

Factor Information

Factor Levels Values

Template2 Manual 1 4 DICE Coefficient, False Negative, False Positive, Jaccard Coefficient

Analysis of Variance

Source DF Adj SS Adj MS F-Value P-Value

Template2 Manual 1 3 2.066 0.68876 14.24 0.000

Error 108 5.223 0.04836

Total 111 7.290

Model Summary

S R-sq R-sq(adj) R-sq(pred)

0.219917 28.35% 26.36% 22.94%

Means

Template2 Manual 1 N Mean StDev 95% CI

DICE Coefficient 28 0.4393 0.2129 (0.3569, 0.5217)

False Negative 28 0.6444 0.2095 (0.5620, 0.7267)

False Positive 28 0.3215 0.2750 (0.2391, 0.4039)

Jaccard Coefficient 28 0.3034 0.1691 (0.2210, 0.3858)

Pooled StDev = 0.219917

**Tukey Pairwise Comparisons**

Grouping Information Using the Tukey Method and 95% Confidence

Template2 Manual 1 N Mean Grouping

False Negative 28 0.6444 A

DICE Coefficient 28 0.4393 B

False Positive 28 0.3215 B

Jaccard Coefficient 28 0.3034 B

Means that do not share a letter are significantly different.

**Tukey Simultaneous 95% CIs**

**Interval Plot of T2M1Value vs Template2 Manual 1**

**One-way ANOVA: T1M2Value versus Template1 Manual 2**

Method

Null hypothesis All means are equal

Alternative hypothesis At least one mean is different

Significance level α = 0.05

Equal variances were assumed for the analysis.

Factor Information

Factor Levels Values

Template1 Manual 2 4 DICE Coefficient, False Negative, False Positive, Jaccard Coefficient

Analysis of Variance

Source DF Adj SS Adj MS F-Value P-Value

Template1 Manual 2 3 0.3312 0.11041 1.89 0.136

Error 108 6.3191 0.05851

Total 111 6.6503

Model Summary

S R-sq R-sq(adj) R-sq(pred)

0.241888 4.98% 2.34% 0.00%

Means

Template1 Manual 2 N Mean StDev 95% CI

DICE Coefficient 28 0.5049 0.2229 (0.4143, 0.5955)

False Negative 28 0.4797 0.2884 (0.3891, 0.5703)

False Positive 28 0.4358 0.2706 (0.3452, 0.5264)

Jaccard Coefficient 28 0.3616 0.1673 (0.2710, 0.4522)

Pooled StDev = 0.241888

**Tukey Pairwise Comparisons**

Grouping Information Using the Tukey Method and 95% Confidence

Template1 Manual 2 N Mean Grouping

DICE Coefficient 28 0.5049 A

False Negative 28 0.4797 A

False Positive 28 0.4358 A

Jaccard Coefficient 28 0.3616 A

Means that do not share a letter are significantly different.

**Tukey Simultaneous 95% CIs**

**Interval Plot of T1M2Value vs Template1 Manual 2**

**One-way ANOVA: T1M1Value versus Template1 Manual1**

Method

Null hypothesis All means are equal

Alternative hypothesis At least one mean is different

Significance level α = 0.05

Equal variances were assumed for the analysis.

Factor Information

Factor Levels Values

Template1 Manual1 4 DICE Coefficient, False Negative, False Positive, Jaccard Coefficient

Analysis of Variance

Source DF Adj SS Adj MS F-Value P-Value

Template1 Manual1 3 0.6458 0.21527 4.88 0.003

Error 108 4.7669 0.04414

Total 111 5.4127

Model Summary

S R-sq R-sq(adj) R-sq(pred)

0.210091 11.93% 9.48% 5.29%

Means

Template1 Manual1 N Mean StDev 95% CI

DICE Coefficient 28 0.4901 0.1834 (0.4114, 0.5688)

False Negative 28 0.5338 0.2407 (0.4551, 0.6125)

False Positive 28 0.3944 0.2516 (0.3157, 0.4731)

Jaccard Coefficient 28 0.3417 0.1471 (0.2630, 0.4204)

Pooled StDev = 0.210091

**Tukey Pairwise Comparisons**

Grouping Information Using the Tukey Method and 95% Confidence

Template1 Manual1 N Mean Grouping

False Negative 28 0.5338 A

DICE Coefficient 28 0.4901 A

False Positive 28 0.3944 A B

Jaccard Coefficient 28 0.3417 B

Means that do not share a letter are significantly different.

**Tukey Simultaneous 95% CIs**

**Interval Plot of T1M1Value vs Template1 Manual1**
